# microRNAs as Novel Therapeutics in Cancer

**DOI:** 10.3390/cancers13071526

**Published:** 2021-03-26

**Authors:** Giulia Romano, Mario Acunzo, Patrick Nana-Sinkam

**Affiliations:** Department of Internal Medicine, Division of Pulmonary Diseases and Critical Care Medicine, Virginia Commonwealth University, Richmond, VA 23298, USA; giulia.romano@vcuhealth.org (G.R.); mario.acunzo@vcuhealth.org (M.A.)

**Keywords:** small non-coding RNA, cancer, therapy

## Abstract

**Simple Summary:**

Over the last few years, we have witnessed incredible advancements in anti-tumor drug development. microRNAs, a class of small non-coding RNAs dysregulated in all cancers, have been recently elected as candidate therapeutics for treating a variety of diseases, including cancer. The scope of this review is to give some insight into the role of the most relevant microRNAs in cancer. We will focus on examining their biological role in tumor development while also providing a broad overview of microRNAs as therapeutics. There is a dedicated focus on the different methods available for microRNA delivery in addition to the efforts being made to increase the specificity of these delivery methods. Finally, we discuss the ongoing clinical trials that are using microRNAs for cancer treatment.

**Abstract:**

In the last 20 years, the functional roles for miRNAs in gene regulation have been well established. MiRNAs act as regulators in virtually all biological pathways and thus have been implicated in numerous diseases, including cancer. They are particularly relevant in regulating the basic hallmarks of cancer, including apoptosis, proliferation, migration, and invasion. Despite the substantial progress made in identifying the molecular mechanisms driving the deregulation of miRNAs in cancer, the clinical translation of these important molecules to therapy remains in its infancy. The paucity of vehicles available for the safe and efficient delivery of miRNAs and ongoing concerns for toxicity remain major obstacles to clinical application. Novel formulations and the development of new vectors have significantly improved the stability of oligonucleotides, increasing the effectiveness of therapy. Furthermore, the use of specific moieties for delivery in target tissues or cells has increased the specificity of treatment. The use of new technologies has allowed small but important steps toward more specific therapeutic delivery in tumor tissues and cells. Although a long road remains, the path ahead holds great potential. Currently, a few miRNA drugs are under investigation in human clinical trials with promising results ahead.

## 1. Introduction

The first small non-coding RNA (sncRNA), the *C. elegans* heterochronic gene lin-4, was identified in 1993 by two independent groups [1,2]. Both teams revealed its ability to regulate lin-4 translation via an antisense RNA-RNA interaction. Only seven years later, Reinhart et al. showed how *C. elegans lin-4* RNA, in combination with heterochronic gene *let-7*, could initiate a temporal cascade of regulatory heterochronic genes [3]. These discoveries have led to a paradigm shift in the understanding of gene regulation, thus uncovering a new important field. Investigators started to focus on the study of these small non-coding RNAs, and many laboratories are currently investing effort and resources in understanding the contribution of these molecules to human disease. In the last three decades, thousands of papers have supported the existence of a class of small ncRNAs termed *microRNAs* (miRNAs) that have biologically relevant roles in gene regulation [4,5]. MiRNAs are defined as short non-coding RNAs~22 nucleotides long, present in all eukaryotic cells, and highly conserved during evolution. Investigators have implicated them in many biological processes, including metabolism, cell cycle, development, differentiation, and apoptosis [6]. MiRNAs contribute to both malignant and benign diseases [6]. It has been estimated that the majority of genes are regulated by miRNAs [7]. As of October 2018, the latest release of miRBase, the official reference knowledge base on miRNAs, the miRNA class of small non-coding RNAs comprised 2658 mature miRNAs in humans, approximately 1978 in mice, 1095 miRNAs in *C. elegans*, and 469 in *Drosophila melanogaster*. The most common mechanism for miRNA targeting in metazoans is based on the complementary match of the miRNA *seed* sequence, which is represented by nucleotides 2–7 from the 5′ end of the miRNA, with canonical sites on the 3′ UTRs (Untranslated Regions) of regulated targets. The miRNA seed is a highly conserved portion of miRNAs and often enables the characterization of miRNA families [8]. There is mounting evidence that miRNAs repress gene expression through translational repression pathways as well as through mRNA degradation [5,9]. Due to the partial complementarity to their targets, miRNAs are capable of targeting multiple genes, often in multiple sites, and some mRNAs have multiple binding sites for different miRNAs [10]. This implies a kaleidoscopic role for a small number of molecules, such as miRNAs, in almost all biological pathways, as well as many diseases, cancer included. In 2002, Calin et al. demonstrated that two miRNA genes, miR-15a and miR-16-1, were present in 13q14, a critical region in chromosome 13 frequently deleted in chronic lymphocytic leukemia (CLL), thus revealing the first association between miRNA deregulation and cancer [11]. This seminal discovery led to a new paradigm in cancer research, demonstrating that alterations in non-coding RNAs can lead to disease. Since this initial discovery, many research laboratories have focused their studies on the miRNA-cancer association, highlighting the fundamental role of these small non-coding RNAs in the development and progression of cancer [12]. For example, in 2006, Costinean et al. showed for the first time that overexpression of a single miRNA, in particular miR-155, could also lead to cancer [13], definitively proving the fundamental role played by these small non-coding RNAs in cancer development. MiRNAs regulate numerous cancer-relevant processes, including apoptosis, proliferation, migration, and invasion, as their ability to target up to several hundred mRNAs supports the concept that aberrant miRNA expression may disrupt a multitude of cell signaling pathways and profoundly influence cancer onset and progression [14,15]. Hundreds of studies have established that miRNA profiles can discriminate between normal and cancerous tissues, discriminate subgroups of tumors, and predict the outcome or response to therapy [16]. MiRNAs have a prominent role in drug resistance [17]. Investigators have successfully demonstrated the employment of miRNAs as sensitizers of tumors to drugs [18,19]. To study the potential direct therapeutic role of miRNAs in vitro and in vivo, researchers either employ synthetic miRNAs, chemically synthesized, double-stranded RNAs which mimic mature endogenous miRNAs, or synthetic anti-miRNA oligonucleotides (also known as AMOs) that are complementary to a mature miRNA which they are designed to neutralize. In this review, we address the most relevant challenges in applying miRNA mimics or anti-miRNAs for directed cancer therapy, from the stabilization of oligonucleotides to specific and safe delivery.

## 2. OncomiRNAs

### 2.1. Tumor Suppressing miRNAs

The term *oncomiRs* [20] refers to miRNAs with a role in cancer as either oncogenes or tumor suppressors (Table 1).

As previously reported, the first tumor suppressor miRNAs (miR-15a and miR16-1) were studied in CLL patients by Calin and colleagues in 2002 [11]. In 2005, the same group characterized the first target of these miRNAs, the antiapoptotic protein Bcl-2 (B-cell lymphoma 2) [21]. Additionally, the *let-7* family members have also been well described as tumor-suppressing miRNAs. Calin et al. in 2004 showed that the 12 members of this family were located in nine different chromosomes, and map to fragile sites associated with different types of solid cancers [23]. *Let-7* family miRNAs target several oncogenes, including Ras, Myc, and HmgA2 (High-mobility group AT-hook 2). Moreover, the *let-7* family is an important marker of fully differentiated cells, being undetectable in stem cells [22]. Another very important group of miRNAs is represented by the miR-34 family, comprised of three differentially expressed members: miR-34a, which is ubiquitously expressed at higher levels, particularly in the brain, and miR-34b/c, which are less expressed, except for in the lung. MiR-34a is encoded by its own transcript, while miR-34b/c share a common primary transcript [24]. These miRNAs are likely to have tissue-specific functions and have been implicated in the p53 pathway. Their expression is induced by p53, and its downstream effect is mediated via targeting of c-Myc, Bcl2, c-Met (hepatocyte growth factor receptor), and Src. Interestingly, miR-34a also plays a fundamental role in the modulation of drug response. For example, in non-small cell lung cancer (NSCLC) cell lines, independent of p53 status (wildtype, mutant or null), miR-34a upregulation induces a downregulation of platelet-derived growth factor receptor-a (PDGFRa) and platelet-derived growth factor receptor-b (PDGFRb), thus restoring TNF-related apoptosis-inducing ligand (TRAIL)-induced apoptosis [25].

The miR-200 family represents another important group of tumor suppressor miRNAs. All five members of this group inhibit epithelial-mesenchymal transition (EMT), migration, invasion, tumor cell adhesion, and metastasis. These miRNAs are transcribed from two different clusters, one located in chromosome 1 (miR-200b/200a/429) and the second in chromosome 12 (miR-200c/141). By targeting vascular endothelial growth factor receptor (VEGFR), one of the most established master determinants of angiogenesis, the miR-200 family has emerged as critical in the regulation of angiogenesis. Moreover, these miRNAs are highly expressed in epithelial tissues, with their targets zinc finger E-box-binding homeobox 1 (ZEB1) and zinc finger E-box-binding homeobox 2 (ZEB2) being well-known markers of EMT. There is a double-negative ZEB/miR200 family feedback loop due to ZEB1′s ability to suppress the expression of miR-200 family members [26].

### 2.2. Oncogenic miRNAs

MiRNAs may also contribute to the initiation and progression of cancers (Table 1). For example, miR-21 is one of the best-described miRNAs upregulated in cancer. It is overexpressed in many types of solid and hematopoietic malignancies, including breast, ovaries, cervix, colon, lung, liver, brain, esophagus, prostate, pancreas, leukemia, and thyroid [27]. Phosphatase and tensin homolog (PTEN), sprouty1 (SPRY1), and 2, reversion-inducing-cysteine-rich protein with Kazal motifs (RECK), and programmed cell death protein 4 (PDCD4) are validated miR-21 targets driving key steps of tumorigenesis, invasion, and metastasis. Moreover, investigators have identified circulating miR-21 as a biomarker for various carcinomas, revealing it as a potential tool for non-invasive diagnosis [28,29,30,31]. The miR-221/222 cluster represents another important example of oncogenic miRNAs. These miRNAs are encoded in the X chromosome in a single transcript, and so they have the same seed sequence, and are highly conserved in vertebrates [32]. Over the last decade, studies have confirmed the overexpression of these two miRNAs in several advanced malignancies, making them two of the most studied miRNAs for diagnostic, prognostic, and therapeutic purposes [3]. A well-known miR-221/222 target is p27/ kinesin-like protein (Kip1), one of the cell cycle inhibitor proteins most downregulated in glioblastoma, thyroid papillary carcinomas, hepatocellular carcinoma, breast, prostate, and pancreatic cancer [32]. Among other miR-221-222 targets, there are Bcl-2-like protein 11 (BIM), PTEN, metalloproteinase inhibitor 3 (TIMP3), forkhead box other 3 (FOXO3), PUMA and estrogen receptor-alfa (ER-α), all crucial components of cell proliferation and apoptosis [32]. Finally, another family of oncogenic miRNAs is the miR-17-92 family, one of the best-characterized polycistronic miRNA clusters, which maps to human chromosome 13 and encodes for six individual miRNAs: miR-17, miR-18a, miR-19a, miR-20a, miR-19b-1, and miR-92a. The first association of this family to cancer was demonstrated in diffuse large B cell lymphomas and B cell lymphoma [34]. Also, deregulation of these miRNAs and their targets have been described in solid tumors such as NSCLC, colon cancer, neuroblastomas, medulloblastoma, and gastric cancer [35]. Cell cycle inhibitors p21/ cyclin-dependent protein kinase (CIP1) and p57/KIP2 are the targets of miR-17 and miR-20 [36]. Furthermore, the expression of the miR-17-92 family was found as an effective predictor of prognosis in different cancers [37].

## 3. The Challenge of Employing miRNAs for Cancer Therapy

The approaches for therapeutic modulation of miRNA expression are two: (1) restoring miRNA activity of tumor suppressor miRNAs or (2) inhibition of oncogenic miRNA function. These two strategies consist of either over-expressing a tumor suppressor miRNA in a tumor tissue where it is downregulated or suppressing an oncogenic miRNA in tumor tissue in which it is overexpressed. Both approaches require cell-specificity and minimal toxicity. There remains a wide gap between in vitro and in vivo applications with many biological barriers, including in vivo nuclease degradation. In vivo, RNAs have very low stability. It has been reported that within 30 min of introduction into murine circulation, miRNAs are cleared from the circulatory system. This is the result of unmodified RNA undergoing degradation by RNAses followed by rapid renal excretion [38]. Moreover, optimization of delivery of miRNAs or antagomiRs requires tissue specificity while maintaining a minimal number of potential off-targets. For tumor-suppressor miRNA replacement to be successful, the approach must also take into consideration that miRNA uptake by cells must achieve physiologically relevant levels.

Another major barrier is the requirement for tissue-specific delivery. In fact, following intravenous administration, only liver and kidney concentrations of miRNA antagonists or miRNA mimics remain high and sustain such levels up to 24 h after injection. In contrast, in all other tissues (brain, heart, lung), miRNA levels decrease quickly [39]. For some tumors, there are also mechanical and biological barriers that hinder the penetration of miRNAs, such as the blood-brain-barrier (BBB) for cancers of the central nervous system or the fibrotic microenvironment in pancreatic cancer. Nonetheless, even if the oligonucleotides were able to reach the intended target tissue, they would most likely be trapped in endosomes and will be transported for endosomes/lysosome degradation, thus being prevented from carrying out efficient gene silencing [39]. Additionally, it is important to consider that these oligonucleotides are often removed by phagocytic immune cells. It has been demonstrated that both single-strand and double-strand oligonucleotides activate the innate immune system response [40] while also being potentially neurotoxic [41]. Finally, we must consider that the interaction of negatively charged and hydrophilic miRNA molecules with the cell membrane would be hindered, resulting in poor cellular uptake. Currently, two miRNA delivery strategies are under development to overcome these obstacles: local and systemic delivery.

### 3.1. Local Delivery

For tumors that are amenable, the best option is local delivery. Teplyuk et al. demonstrated that intracranial injections of miR-10b antisense led to target downregulation and attenuated growth and progression of established glioblastoma multiforme (GBM) [42]. There are also convincing examples of topical skin cancer treatment with oligonucleotides that validate the possibility of using miRNAs in treating this type of cancer [43]. Inoue et al. demonstrated that using a topical ointment containing miR-634 inhibited in vivo tumor growth without toxicity in two different skin tumor models: a cSCC xenograft mouse model and 7,12-dimethylbenz[a]anthracene (DMBA)/12-O-tetradecanoylphorbol-13-acetate (TPA)-induced papilloma mouse model [44].

Trang et al. have shown that intranasal delivery of anti-let-7 enhances lung tumor formation in vivo. This represents an important observation that naked small interfering RNAs can be successfully delivered to the lung via the intranasal passage while maintaining stability [45].

Unfortunately, very few tumors can be treated in this manner, including primary and well-localized tumors. The treatment of advanced metastatic tumors must be accomplished through systemic delivery. Nonetheless, there many different strategies that have been developed in recent years to overcome the challenges faced by systemic delivery.

### 3.2. Systemic Delivery

**Figure 1:**
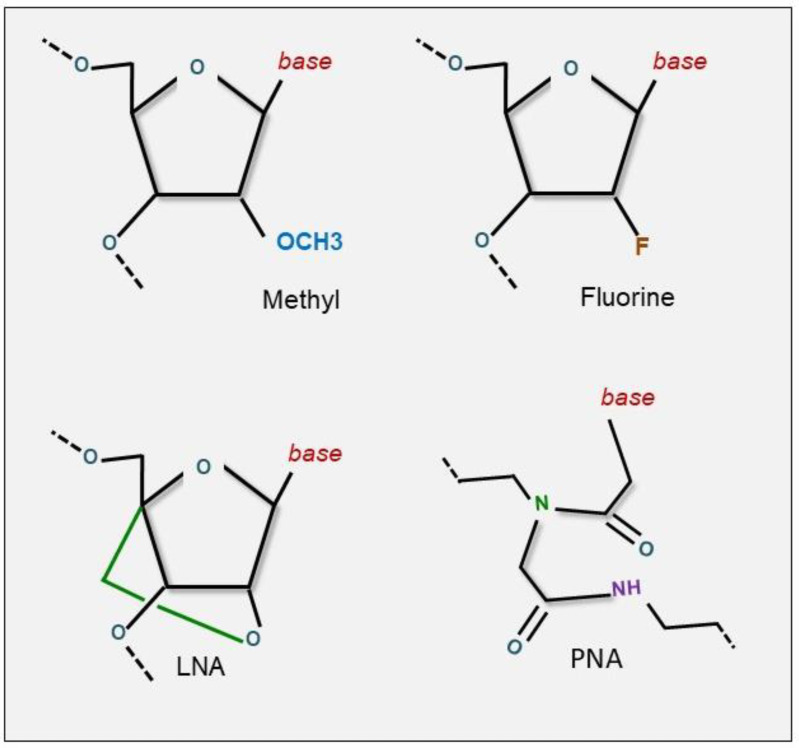
Different modifications to optimize oligonucleotides delivery. chemical modification of 2′-OH ribose with fluoro or methyl group results in oligonucleotide stabilization improving resistance to degradation in plasma. anti-miRNAs modified with LNAs (Locked Nucleic Acid) are more stable and have a high affinity with target miRNA. PNAs have their affinity to RNA than DNA and are very resistant to DNAses and proteases.

As previously mentioned, one of the most important challenges for systemic delivery is the improvement of oligonucleotide stability [46] while decreasing the innate immune response [47]. To address this challenge, many approaches have emerged, the easiest being the development of stable molecules through chemical modifications on the 2′-OH ribose with a fluoro, ammino, or methyl group (Figure 1).

It has been shown that these modified molecules are 1000-fold more resistant to degradation in plasma than their un-modified RNA counterparts [48]. Unfortunately, these modified oligonucleotides are often rapidly degraded in serum [49]. Increased stability has been achieved by altering the passenger strand of double-strand miRNA mimics. For example, Akao et al. modified the sequences of the passenger strand in the miR-143 duplex by applying a chemical modification at the 3′-overhang portion of the miRNA, leading to increased activity and stability against nucleases [50]. Another chemical modification that is used to stabilize oligonucleotides consists of the employment of Locked Nucleic Acid (LNA) oligonucleotides where an extra bridge connects the 2′ oxygen with the 4′ carbon for “locking” the ribose ring in the ideal conformation for Watson-Crick binding [51] (Figure 1). In 2003, this technique was already found to be effective, demonstrating the efficacy of LNAs in tumor growth inhibition in vivo [52]. Di Martino et al. analyzed LNA- i-miR-221 pharmacokinetics and pharmacodynamics in NOD.SCID mice and Cynomolgus monkeys (*Macaca fascicularis*) plasma, urine, and tissues, and found that they showed a short half-life, optimal tissue bio-viability, minimal urine excretion of LNA-i-miR-221, and no toxicity [53]. Miravirsen (SPC3649, Roche, (Basel, Switzerland)), a locked nucleic acid-modified phosphorothioate oligonucleotide targeting miR-122, is undergoing multiple phase II clinical trials for the treatment of chronic hepatitis C patients, having already been proven to be safe and effective long-term [54,55]. Furthermore, the same group demonstrated that this anti-miRNA is specific for miR-122. They indeed demonstrated that only miR-122 expression level in plasma is decreased in patients following treatment [55]. An alternative modification strategy consists of creating peptide nucleic acids (PNAs), which are artificially synthesized uncharged oligonucleotides that display a higher affinity to RNA than to DNA, and are resistant to DNases and proteases (Figure 1). Bcl-2 and c-Myc were successfully downregulated by specific antisense PNAs [56,57]. Currently, there are several studies that have successfully employed anti miRNA PNAs in cancer therapy: PNA-antimiR-21 inhibited tumor growth in vivo in a breast cancer model [58]; miR-155 oncomiR was inhibited in a mouse model of lymphoma by PNA antimiRs attached to a peptide with a low pH-induced transmembrane structure (pHLIP), this conjugation also has the advantage of evading systemic clearance by the liver and further facilitates cell entry via a non-endocytic pathway [59]. In aggressive breast cancer cell lines, it was shown that polyarginine-PNA conjugated anti-miR-221 was specific for miR-221, and demonstrated efficient cellular uptake without the aid of transfection reagents [57]. Segal et al. found that hydrophobically modified miRs (hmiR) added directly to culture medium or subcutaneously can enhance the biodistribution of the miRNAs in NSCLC [60].

## 4. Overview of Delivery Systems

### 4.1. Vectors

**Figure 2:**
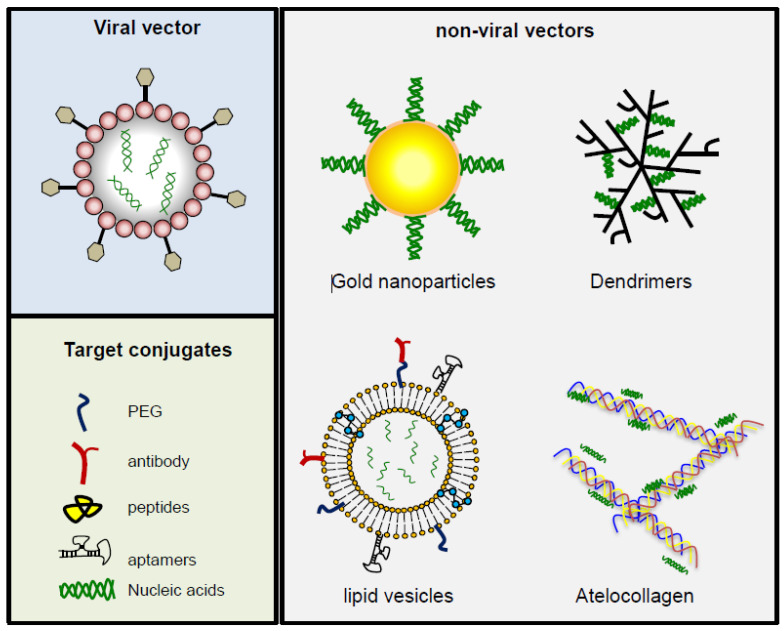
Schematic description of delivery shuttles developed to increase the stability and efficiency of systemic delivery of oligonucleotides. Viral vectors are very efficient but immunogenic. Non-viral vectors are less immunogenic but have faulty inefficiency, toxicity, and lack specificity. Different strategies have been developed to overcome these obstacles. Conjugation with PEG makes these vectors more stable and, to enhance specificity, they can be combined with peptides, antibodies, or peptides that recognize a target on the cell surface.

In order to address poor cellular uptake due to charge repulsion between miRNAs and the cell membrane, some vector-mediated delivery systems have been develope, two of which are currently in use: viral and synthetic delivery systems. Synthetic systems are less efficient but simple to manufacture, have tolerance for cargo sizes, and are less immunogenic [61] (Figure 2).

#### 4.1.1. Viral

Viral delivery of synthetic miRNA has proven to be very efficient. Adenoviruses (AVs), lentiviruses (LVs), and adeno-associated viruses (AAVs) have been employed with miRNAs in various cancer models. Interestingly, for more specific uptake of oligonucleotides by cancer cells, a capside protein can be engineered. This type of delivery in a murine liver cancer model has yielded very good results [62]. Unfortunately, the main problem for adenoviruses and adeno-associated viruses remains the immunogenicity and the transient nature of miRNA expression, while lentiviruses present a genomic integration safety hazard [63].

#### 4.1.2. Non-Viral

Table 2 summarizes the most employed non-viral vectors. The most used in-vitro transfection reagents are lipid-based nanoparticles [64]. Some of these have yielded very good results for intratumoral and systemic delivery in head and neck squamous cell carcinoma (hnscc), nsclc, lymphoma, breast, and pancreatic cancers [39]. Cationic lipoplexes are less utilized, given that they tend to interact with serum proteins, reducing their half-lives. Furthermore, this protein complex can stimulate immune recognition and trigger elimination by the reticuloendothelial system (RES). However, generally neutral and anionic carriers need to have a cationic core to bind the negatively charged miRNAs. By employing polyethylenimine (PEI), it is possible to create a stable biocompatibility complex with miRNAs. The polyplexes formed by PEI and nucleic acids harbor a net positive charge, and the interaction with the negatively charged cell membrane is thus favored. Hwang et al. delivered miR-124a, a neuron-specific miRNA that promotes neurogenesis, to neurons in vivo thus, crossing the blood-brain-barrier. They conjugated PEI with rabies virus glycoprotein (RVG) that specifically bound the nicotinic acetylcholine receptor. By injecting the miR-124a/RVG-SSPEI via tail vein, they demonstrated an enhanced accumulation of miR-124a in the isolated brain [65]. A strategy for improving biocompatibility and the stability of lipoplexes is PEGylation (attachment of polyethylene glycol (PEG) polymer chains to the vesicles). It has been shown that this modification increases persistence in circulation for up to 72 h, thus allowing for greater accumulation in the affected site [66]. Employment of dendrimer-encapsulated nanoparticles (DENs) is another attractive delivery system. DENs are synthesized by a template approach using dendrimers that are repetitively branched molecules. A recent study demonstrated a pronounced survival benefit in an aggressive preclinical genetic cancer model using dendrimers to deliver a let-7g mimic. The authors tested such a mimic in chronically ill mice bearing MYC-driven tumors and found inhibition of tumor growth and dramatically extended survival [67]. Poly (amidoamine) (PAMAM) dendrimers have also been applied for drug, gene and, siRNA delivery in cancer therapy [68].

The poly lactic-*co*-glycolic acid (PLGA) is an FDA-approved synthetic copolymer that has been used to fabricate devices for drug delivery and tissue engineering applications in the past two decades. PLGA has been extensively studied for the development of methods for controlled delivery of small molecule drugs, proteins, and other macromolecules. Specifically, PLGA is employed as a drug delivery device for Lupron Depot, a synthetic hormone used in the treatment of advanced prostate cancer. PLGA is biocompatible and biodegradable, making it a very good candidate for delivery of miRNAs. In addition, it can protect nucleic acids from nuclease degradation, and it has thus been used for successful delivery of DNA and RNA, with some ongoing clinical trials using this polymer demonstrating great potential [69]. Investigators applied PLGA in a (miR-155)-dependent mouse model of lymphoma. Both systemic and efficient delivery of PLGA nanoparticles encapsulating antisense peptide nucleic acids inhibited miR-155 and slowed tumor growth [70]. Two recent studies have shown how PLGA may be used as a vehicle for synthetic miRNAs delivery in vitro and in vivo with very good results. In the first one, the authors used PLGA-PEG nanoparticles to deliver antisense-miR-21 in combination with the drug (orlistat) for the treatment of triple-negative breast cancer, finding a significantly enhanced apoptotic effect in vitro in MDA-MB-231 and SK-BR-3 triple-negative breast cancer (TNBC) compared to normal breast fibroblast cells [71]. In the second study, the authors, studying the fusion of macrophages to form foreign body giant cells (FBGC), developed a method for in vivo delivery of a miR-223 mimic utilizing PLGA nanoparticles. After demonstrating the efficiency of the nanoparticles in targeting implant-adherent cells, they are also proved that the delivery and overexpression of miR-223 decreased FBGCs in vivo [72].

Furthermore, naturally occurring polymers, such as chitosan, protamine, atelocollagen, and peptides from a translocation domain, can be used as a delivery system. miR-16 conjugated to atelocollagen injected into tail veins of mice reduced bone metastases in a prostate cancer xenograft model [73]. In bone-metastatic prostate tumors, efficient delivery of miR-15a and miR-16-1 in vivo can be achieved along with an increase in anti-cancer efficacy compared to other treatments in vivo [74]. In esophageal squamous cell carcinoma, investigators showed that atelocollagen prolonged the accumulation of miRNA-375 by using fluorescently-labeled miRNAs and an in vivo imaging system [75].

Protamine is an FDA-approved, naturally occurring peptide of ∼5000 Da obtained from the sperm of salmon and certain other species of fish. In an interesting study aimed to improve the delivery efficiency of miR-145 to cancer cells, the authors optimized a liposome-based delivery system using protamine as a DNA-condensing agent to form liposome-protamine-DNA (LPD) ternary complexes. The LPD complex showed an increase in transfection efficacy and a decrease in cell toxicity [76]. The biggest disadvantages of protamine are thromboxane generation and immunological reactions. In recent years, to reduce immune toxicity mediated by native protamine, several low molecular weight protamines (LMWP) have been synthesized for siRNA delivery. Suh et al. used LMWP to deliver miR-29b targeting anti-osteogenic factor gene expression in stem cells to promote osteoblastic differentiation. They found that mRNA levels of all osteogenic markers increased at 48 h, which was higher than that observed using lipoplex delivery systems for the same miRNA [77]. *S*table-*N*ucleic-*A*cid-*L*ipid-*P*articles (SNALPS) are 120-nanometer biopolymers characterized by high vesicle loading, good transfection efficiency, and stability in serum. In 2014, Di Martino et al. showed that SNALPS carrying miR-34a repressed multiple myeloma (MM) cell growth in vitro and in vivo (MM xenografts in SCID mice) [78]. In 2015, Costa et al. demonstrated the efficacy of SNALP-formulated anti-miR-21 oligonucleotides against glioblastoma in vitro and in vivo, confirming the high potential of this carrier [79]. Lastly, inorganic materials have been employed as vectors for the delivery of small oligonucleotides, with silica-based nanoparticles and gold nanoparticles as the most used inorganic materials. Recently, Silica nanoparticles, conjugated with a neuroblastoma specific antigen (disialoganglioside, GD_2_), were used to deliver miR-34a successfully. The authors assessed the delivery specificity and the up-regulation of miR-34a in neuroblastoma cell lines compared to HEK293, demonstrating that anti-GD2-miR-34a-NPs were effective in the reduction of neuroblastoma in mice [80]. This approach has been employed as a single-vehicle system for cellular delivery of a miR-122 antagomir as well as hydrophobic small-molecule inhibitors using mesoporous silica nanoparticles. Investigators have successfully delivered anti-miR-122 and a compound (sm122) which inhibited endogenous miR-122 by effectively blocking the synthesis of its conjugated pri-miRNA through the same vehicle system in Hepatocellular Carcinoma cells (HCCs) Huh7, obtaining good intracellular stability, efficient cellular uptake/endosomal escape, and target-triggered release of drugs results [81]. Gold (Au) nanoparticles (AuNPs) exhibit low toxicity and immunogenicity, and given their physical, chemical, optical, and electronic properties, they have been used for diagnostic purposes [82]. Investigators have developed a method for delivering unmodified miRNAs into cells using cysteamine-functionalized AuNPs. Amino-functionalized gold nanoparticles coated with PEG were complexed with antimiR-31 and miR-1323 and tested on four different cell lines of two different types of cancer, including neuroblastoma (NGP and SH-SY5Y) ovarian cancer (OVCAR8 and HEYA8). The authors demonstrated good release with the noncytotoxic effect [83].

Near-infrared-radiation (NIR) responsive hollow gold nanoparticle (HGNPs) to deliver a miR-21 inhibitor coupled with doxorubicin (Dox) resulted in a subsequent release for both while achieving synergistic efficacy. The authors tested this methodology on MDA-MB-231, MCF7 breast cell lines, MCF-10 A, and MDA-MB-231-derived stem cells. They delivered anti-miR-21 to sensitize cancer cells to doxorubicin and, as the second step, released the drug, enhancing anti-cancer efficacy by 8-fold and increasing anti-cancer stem cell activity by 50-fold. Through intravenous administration of the same compound in a MDA-MB-231 xenograft mouse model, they showed high tumor accumulation and significantly improved efficacy, 4-fold compared to the free doxorubicin group [84]. EnGeneIC Ltd (Lane Cove West, Australia) developed an alternative carrier based on bacterially derived nanocells (EDV™ nanocells) [85]. This system has been used by Reid et al. to develop TargomiR, a miRNA mimic-based treatment for recurrent thoracic cancer [86]. It led to a phase 1 MesomiR-1 (miR-16 mimic) trial (NCT02369198) on mesothelioma patients, which was completed with good results in April 2017; 22 of 26 patients had a therapeutic response that lasted 32 weeks [87].

#### 4.1.3. Extracellular Vesicles

Extracellular Vesicles (EVs) are emerging as potential vehicles for miRNA delivery [88,89,90]. EVs represent a variety of natural vesicles produced by all cells, differing by size and biogenesis pathway [91]. They can be used as a “natural” delivery system. In the last decade, many groups are working on delivery system by leveraging on some of the characteristics of EVs. For example, CD47 is a “don’t eat me” signal that, if present on membranes, can protect the cells and EVs from phagocytosis by monocytes and macrophages [92]. EVs engineered for therapy can utilize CD47 to increase their life span [93]. Furthermore, Hoshino et al. found an association between some integrins present on EVs membranes and tissue metastasis: the presence of α_6_β_4_ and α_6_β_1_ was associated with lung metastasis, while exosome integrin α_v_β_5_ was linked to liver metastasis [94] This information can be used to “create” tissue-specific EVs increasing the delivery specificity. Moreover, EVs can cross the BBB, thus opening a new avenue for treatment of brain tumors or brain metastasis [95].

## 5. Cell and/or Tissue Specificity 

Once an optimal delivery method allowing sufficient oligonucleotide stability is developed, cell and tumor delivery specificity remains crucial to successful therapy. In recent years, hepatic delivery methods have yielded encouraging results, as well as interesting reports of extra-hepatic targeting [96]. The four most studied targeted conjugates for targeted delivery to particular cancer types are glycoconjugates, peptides, aptamers, and antibodies.

### 5.1. Glycoconjugates

Glycoconjugates are involved in cell-cell interactions, including cell-cell recognition as well as cell-matrix interactions. For effective hepatic delivery to liver cells, investigators have used asialoglycoprotein receptor (ASGR), an endocytotic cell surface receptor expressed by hepatocytes. One of the first studies on miRNA delivery leveraging ASGR used lactosylated gramicidin-containing lipid nanoparticles (Lac-GLN), including AGSR ligand N-lactobionyl-dioleoyl phosphatidylethanolamine, which were capable of effectively delivering anti-miRNA-155 into SK-Hep-1 and HepG2, human hepatocellular carcinoma cells, leading to upregulation of two of the most important miR-155 targets (C/EBPβ and FOXP3 genes). Intravenous injection of 1.5 mg/kg of anti-miR-155 resulted in preferential accumulation of anti-miR-155 in hepatocytes and an up-regulation of C/EBPβ and FOXP3 [97]. Researchers at Alnylam Pharmaceuticals (Cambrige, MA, USA) developed a siRNA conjugated multivalent N-acetylgalatosammine (GalNac), showing very good uptake in primary murine hepatocytes following subcutaneous administration into murine liver [98]. GalNac was also used to deliver Antisense oligonucleotides (ASOs) with a 10-fold improvement in liver uptake in mice. The observed increase for ASOs was lower than that observed for siRNAs, despite efficient accumulation in the liver [99]. There are few GalNAC-siRNA in clinical development studies for their safety profile [100]. Additionally, Phase 3 studies are also now in progress for siRNA glycoconjugate Fitusirian (ALN-AT3SC) for the treatment of hemophilia A and B (NCT03417102, NCT03549871, NCT03417245. Clinical Trials.Gov) [101,102]. The results of these clinical trials may lead to the employment of this reagent for the delivery of oligonucleotides to the liver.

### 5.2. Peptides

The most studied peptides for oligonucleotide delivery are integrin ligands, such as cyclic Arg-Gly-Asp (RGD). RGD peptides are employed in association with a variety of carrier types (lipoplexes, dendrimers, and other polymers) [96]. Insulin-like growth factor 1 was used for siRNA delivery in vitro in human breast cancer cell lines MCF7 [103], but no in vivo studies have been published since this initial observation. In the previous section, we mentioned how Suh et al. employed an arginine-rich CPP to deliver miR-29b targeting anti-osteogenic factor gene expression in stem cells to promote osteoblastic differentiation [77]. The amphiphilic R3V6 peptide is a good carrier in vitro and in vivo for the delivery of anti-miR-21 in glioblastoma. The authors recorded low tumor growth following intratumoral injection of the antimiR-21/R3V6 complex, compared with the antimiR-21/PEI25k and scrambled-antisense/R3V6 compounds [104].

### 5.3. Aptamers

Aptamers are short structured single-stranded DNA or RNA (ssDNA or ssRNA) that can bind to pre-selected targets, including proteins and peptides with high affinity and specificity. They can be developed against almost any protein target, including transmembrane receptors, by a combinatorial strategy termed Systematic Evolution of Ligands by EXponential enrichment (SELEX) [105].

In 2009, a pioneering study on the use of aptamers for oligonucleotide delivery used advanced prostate-specific membrane antigen (PSMA) to systemically deliver aptamer-siRNA chimeras to target PLK1 in athymic mice, leading to pronounced regression of PSMA-expressing tumors [106]. Researchers used an aptamer-miRNA conjugate for the delivery of the miRNA tumor suppressor let-7g in both in vitro and in vivo models of lung cancer using the anti-Axl GL21.T aptamer. The authors showed selective delivery to target cells by using A549 Axl^+/+^ cells with MCF7 Axl^−/−^ as a negative control. This led to reduced tumor growth in a xenograft model of lung adenocarcinoma following tail vein administration [107]. Recently, researchers have developed therapeutic RNA nanoparticles containing anti-miR-21, with epidermal growth factor receptor (EGFR) targeting aptamers to internalize RNA nanoparticles into cancer cells via receptor-mediated endocytosis. These nanoparticles have been employed against the TNBC cell line MDA-MB-231. Upon obtaining good results with the downregulation of miR-21 in vitro, they tested the nanoparticles in vivo via tail vein injection into orthotopic TNBC tumor-bearing mice, showing that the nanoparticles were RNase resistant, thermodynamically stable, remaining intact and strongly bound to tumors with little or no accumulation in healthy organs 8 h post-injection [108]. Kardani et al. demonstrated the efficient and specific delivery of anti-miR-155 in breast cancer cells using AuNPs and nucleolin-specific aptamer nanocarrier [109].

### 5.4. Antibodies

The capacity for antibodies to bind specific cell surface receptors has been exploited in order to design a specific vehicle for oligonucleotide delivery. Monoclonal antibodies have been recently employed in various studies. One group designed an integrin αvβ3-targeted nanoparticle to selectively deliver anti–miR-132 to the tumor endothelium of mice. Downregulation of miR-132 led to the upregulation of its target p120RasGAP, which was expressed in normal but not tumor endothelium. Systemic administration of anti–miR-132 nanoparticles not only blocked angiogenesis but also significantly decreased tumor burden and angiogenesis in an orthotopic xenograft mouse model of human breast carcinoma [110]. Another group developed a liposome-polycation-hyaluronic acid (LPH) nanoparticle formulation modified with a tumor-targeting single-chain antibody fragment (scFv) for systemic delivery of siRNA (against c-Myc, MDM2, and VEGF) and miR-34a into experimental lung metastasis of murine B16F10 melanoma [111]. An scFv-protamine chimera targeting Her2 was used to deliver growth-inhibitory siRNAs to Her2 positive breast cancer cells causing retardation of tumor growth in an orthotopic breast cancer model [112]. Another study illustrated in the already mentioned paper by Tivnan et al. employed silica nanoparticles conjugated with a neuroblastoma specific antigen (disialoganglioside), GD_2_ [80]. However, since then, few papers have been published using antibody-conjugated nanoparticles, indicating that there are complexities to be addressed. Several studies showed good delivery to cells, such as blood cells displaying a well-characterized membrane biomarker. Meissner et al. conducted both in vitro and in vivo studies by developing liposomes with an antisense core (siBCL2) complexed by either a cationic lipid, 1,2-dioleoyl-3-trimethylammonium propane (DOTAP), or a synthetic polycation, polyethyleneimine, encapsulated within liposomes modified with polyethylenoglycol; the liposomal shells were enriched with covalently-bound antibodies recognizing CD20 [113]. (Table 3).

In 2020, Su et al. conjugated a scavenger receptor/Toll-like receptor 9 agonist (CpG1668 oligonucleotide) to a miR-146a mimic oligonucleotide (C-miR146a), enhancing the internalization and the delivery to the cytoplasm of target myeloid cells and leukemia cells [114].

## 6. Off-Target Effects

Lastly, an important consideration in the development of any therapeutic is the off-target effects of using oligonucleotides for miRNA therapy. After illustrating the inflammatory response through the activation of Toll-like receptors triggered by oligonucleotides and/or their delivery vehicles, it important to note that such processes can be mitigated but not fully eliminated through chemical modification such as the substitution of the 2′ position of ribose with 2′-*O*-methyl, 2′-fluoro, 2′-deoxy or a locked nucleic acid. Furthermore, exogenous siRNAs can saturate the endogenous RNAi machinery, causing widespread effects on miRNA processing and function [115]. It is also very important to consider the nature of miRNAs. Sequence seed complementarity is of utmost importance for miRNA target recognition. Concurrently, a very important characteristic lies in the capacity for multiple targets and simultaneous modulation of multiple biological pathways [12].

**Figure 3:**
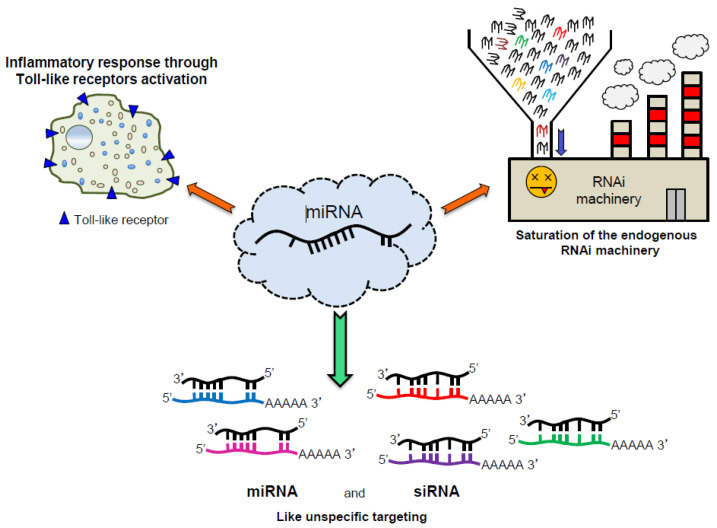
Schematic representation of effects due to exogenous oligonucleotides overexpression. The microRNA overexpression can trigger the inflammatory response through the activation of the Toll-like receptors (TLRs) (**up-left**), can saturate the RNAi machinery inhibiting the processing (**up-right**), and can induce unspecific targeting inducing unwanted off-targeting effect (**bottom**).

This characteristic represents a limitation in the design of therapeutic miRNAs. Indeed, one of the most important issues facing synthetic non–coding RNAs such as siRNA and miRNAs in therapy as well in basic research is the off-targeting effect [116,117]. It has been reported that a partial and non-specific matching between a miRNA and an mRNA sequence could lead to mRNA degradation. This “off-targeting” effect becomes a significant issue when small non-coding RNA therapy is attempted, considering that the effects of the delivered miRNA could be diluted by other unspecific targeting and at the same time could induce undesirable side effects [118]. Recently, significant efforts have been made to predict the off-targeting effects of synthetic miRNAs [119,120] (Figure 3).

## 7. miRNA Drugs in Clinical Trials

There are some miRNA drugs under investigation in clinical trials in cancer and in other diseases (see Table 4).

### 7.1. Cancer

Mirna Therapeutic’s (now Synlogic, city, state abbrev if USA, country) MRX34 is a double-stranded RNA mimic of tumor suppressor miR-34 encapsulated in a liposomal nanoparticle. miR-34 represents the first miRNA mimic to enter clinical trials and has demonstrated compelling clinical results as a single agent therapy, including confirmed partial responses in patients with renal cell carcinoma, acral melanoma, and hepatocellular carcinoma, with phase I trial that should have ended in December 2016. Unfortunately, the study was halted after severe adverse events (SAE), resulting in four patient deaths [121].

The previously mentioned MesomiR-1 (a miR-16 mimic delivered by targeted bacterial minicells) (NCT02369198) from ENGeneIC was tested in a now completed phase 1 clinical trial on mesothelioma patients [87]. An additional agent, MRG-106 (Cobomarsen), an LNA anti-miR-155, is currently in Phase 1 (NCT02580552) and 2 (NCT03713320, NCT03837457) clinical trials for treating lymphoma and leukemia [122,128]. In early 2019, Regulus Therapeutics Inc. (Carlsbad, CA, USA) announced a new candidate, RGLS5579, an anti-miR-10, for treatment of glioblastoma multiforme, but currently, it remains in preclinical phase.

### 7.2. Other Disease

The already cited LNA-modified-anti-miR-122 (SPC3649) is in multiple phase II clinical trials for the treatment of chronic hepatitis C patients [54,55]. MRG-201 (Remlarsen) by MiRagen Therapeutics (Boulder, CO, USA) is an LNA miR-29 mimic in phase 2 clinical trial (NCT03601052) that can limit the formation of fibrous scar tissue in the treatment of cutaneous fibrosis or idiopathic pulmonary fibrosis [123]. MRG-110 an LNA anti-miR-92-3p for the treatment of ischemic conditions is now in phase 1 (NCT03603431) [124]. Regulus Therapeutics Inc has already had two other products in clinical trials: (1) RG-012, an anti-miR-21 drug for the treatment of Alport syndrome that completed phase 1 (NCT03373786) in April 2019, and (2) RGLS4326 an anti-miR17 for autosomal dominant polycystic kidney disease (ADPKD) treatment that is currently on partial clinical hold by the U.S. Food and Drug Administration [125].

Finally, Abivax (Paris, France) is producing and testing ABX464, a REV (HIV protein) inhibitor, in different diseases. It has been shown that ABX464 can not only inhibit viral replication but can upregulate the anti-inflammatory response through upregulating miR-124 [126,127]. Currently, seven phase 1 and 2 clinical trials using ABX464 have closed, demonstrating encouraging results, and six-phase 2 and 3 remain open. ABX464 has been used to treat several diseases ranging from HIV and Covid-19 to ulcerative colitis, Crohn;s Disease, and rheumatoid arthritis (clinicaltrials.gov). Moving forward, the results of these clinical trials will play a key role in the improvement of cancer therapy in the future.

## 8. Conclusions

In the past two decades, miRNAs have emerged as being important in the development and progression of cancer as well as drug resistance. Despite the substantial progress made in the understanding of the molecular mechanisms underlying the deregulation of miRNAs in cancer, they have yet to be fully translated to therapeutics. Some miRNAs, such as miR21, miR34, and let-7, have been used as biomarkers [22,24]; nonetheless, we are still far from being able to employ miRNAs for the cure of diseases such as cancer, with the major obstacle remaining their effective delivery while minimizing off-target effects. Despite the great effort made worldwide to improve various techniques, current delivery methods have yet to be optimized, with each having distinct advantages and disadvantages. Viral-based delivery is very efficient but carries an elevated activation of the immune system.

Conversely, current non-viral-based vectors are fairly well-tolerated by the immune system, but have faulty inefficiency, toxicity, and lack of specificity. All non-viral delivery carriers possess a cationic surface to take advantage of the miRNA’s negative charge for packing. Increasing the number and density of amine improves the efficacy of transfection but leads to increased cytotoxicity. As opposed to local delivery, systemic delivery still requires extensive optimization. Novel improvements to reduce cytotoxicity could be achieved through the development of new biocompatible materials with the capacity for conjugating cationic vectors or through the employment of biodegradable nanoparticles. Additional biological barriers may prevent efficient cellular intake of the miRNAs, which in turn leads to poor target efficiency. Other external factors including ligands (e.g., hyaluronic acid and folate acid), targeting peptides (e.g., cRGD and RVG), aptamers (e.g., GL21.T and AS1411), antibodies (e.g., scFv, GD2), and other molecules that enhance active targeted delivery are currently being investigated to better direct nanoparticles (NPs) to specific organs or cells.

In order to minimize the off-target effects, an interesting approach would be to leverage the natural properties of endogenous miRNAs, which target multiple genes, often in multiple sites, due to the partial complementarity they exhibit to their targets. A recent paper showed that artificial miRNA (a-miRs) can successfully repress at least two targets simultaneously by binding to one or more sites in their 3′ UTRs [8]. It is thus, possible to simultaneously and efficiently downregulate multiple proteins in the same pathway, reducing the off-target effects. The field of miRNA therapeutics while growing remains in the early stages, with many investing resources into the development of miRNA mimics and antagomirs. Continued efforts to overcome the challenges inherent in miRNA therapy will soon be rewarded with the development patient-specific miRNA mimics or antimiRs with a goal of effective personalized cancer therapy.

## Figures and Tables

**Table 1:** A schematic overview of oncomiRs and tumor-suppressor miRNAs.

miRNA	Target	Ref.
Tumor suppressor		
miR-15a/miR-16	Bcl-2	[11,21]
let-7 family	Ras, Myc, HmgA2	[22,23]
miR-34 family	c-Myc, Bcl2, c-Met, Src	[24,25]
miR-200 family	VEGFR, ZEB1, ZEB2	[26]
OncomiRs		
miR-21	PTEN, Sprouty1 & 2, Reck	[27,28,29,30,31]
miR-221/miR-222	p27/kip1, Bim, PTEN TIMP3, FOXO3, PUMA, ER-α	[32,33]
miR-17-92 family	p21/CIP1, p57/KIP2	[34,35,36,37]

**Table 2:** Most employed non-viral vectors.

Non-Viral Nanoparticles	Pos./Neg.	Study	Ref.
Lipid-based nanoparticles			
polyethylenimine (PEI)	good biocompatibility	miR-124 in neurons	[65]
dendrimer-encapsulated nanoparticles (DENs)	versatility	let-7g MYC-driven tumors	[67]
poly lactic-co-glycolic acid (PLGA)	Biocompatible biodegradable	miR-155-dependent mouse lymphoma; anti-miR-21 breast, miR-223	[70,71,72]
Natural polymers			
atelocollagen		miR-15 and miR-16 in prostate cancer miR-375 esophageal carcinoma	[73,74,75]
low molecular weight protamines (LMWP)		miR-29b in osteoblasts	[77]
stable-nucleic-acid-lipid-particles (SNALPS)	very stable in serum	miR-34a in MM; miR-21 GBM	[78,79]
Inorganic material			
silica-based nanoparticles		miR-34 neuroblastoma, anti-mir-122 in hepatocellular carcinoma	[80,81]
gold (Au) nanoparticles (AuNPs)	low toxicity and immunogenicity	anti-miR-31and miR-1323 in neuroblastoma and ovarian; miR-21 and doxorubicin in breast	[83,84]

**Table 3:** Scheme of targeted conjugates for targeted delivery.

Target Conjugates	Study	Ref.
Glycoconjugates		
Asialoglycoprotein receptor (ASGR),	anti-miR-155 hepatocellular carcinoma	[97]
N-Acetilgalattosammine (GalNac)	miR-155 hepatocytes; ASOs and siRNAs phase1-2-3 studies	[98,99,100,101,102]
Peptides		
Insulin-like Growth Factor 1	delivery siRNA in breast	[103]
Arginine-rich CPP	miR-29b in osteoblasts	[77]
R3V6 peptide	anti-miR-21 in glioblastoma	[104]
Aptamers		
Axl GL21.T	let-7g in lung cancer	[107]
EGFR-target aptamers	anti-miR-21 in breast	[108]
Nucleolin	Anti-miR-155	[109]
Antibodies		
integrin αvβ3-targeted	antimiR-132 breast model	[110]
scFv	siHer2 in breast	[112]
GD2	miR-34 in neuroblastoma	[80]
CD20	siBCL2	[113]

**Table 4:** Clinical trials using miRNAs or anti-miRNAs.

Drug	miRNA/antimiRNA	Disease	Clinical Trial	REF
Cancer				
MRX34	miR-34 mimic	Renal Cell Carcinoma	ended	[121]
MesomiR-1	miR-16 mimic	Mesothelioma	phase 1 Completed	[87]
MRG-106 Cobomarsen	LNA anti-miR-155	lymphoma, leukemia	Phase 1 & 2	[122,123]
Other Disease				
MRG-201 Remlarsen	LNA miR-29 mimic	cutaneous fibrosis and idiopathic pulmonary fibrosis	phase 2	[123]
Miravirsen (SPC3649)	LNA-modified-anti-miR-122	chronic hepatitis C	Phase 2	[54,55]
MRG-110	LNA anti-miR-92-3p	ischemic conditions	phase 1	[124]
RG-012	anti-miR-21	Alport syndrome	phase 1	
RGLS4326	anti-miR-17	Autosomal Dominant Polycystic Kidney Disease	clinical hold	[125]
ABX464	upregulate miR-124	HIV, Covid-19, Ulcerative Colitis, Crohn Disease, and Rheumatoid Arthritis	Phase 2 & 3	[126,127]

## Data Availability

Not applicable.

## Abbreviations

**Abbrv.**	**Definition**
AAVs	Adeno-Associated Viruses
ADPKD	Autosomal Dominant Polycystic Kidney Disease
AMOs	anti-miRNA Oligonucleotides
ASGR	Asialoglycoprotein Receptor
ASOs	Antisense Oligonucleotides
AuNPs	Gold (Au) nanoparticles
Avs	Adenovirus
BBB	Blood-Brain-Barrier
BCL2	B Cell lynphoma 2
BIM	Bcl-2-like protein 11
CIP	cyclin-dependent protein kinase
CLL	Chronic Lymphocytic Leukemia
c-MET	hepatocyte growth factor receptor
cSCC	cutaneus Squamous Cell Carcinoma
DENs	Dendrimer-Encapsulated Nanoparticles
DOTAP	1,2-Dioleoyl-3-trimethylammonium propane
Dox	Doxorubicin
EMT	Epithelial-Mesenchymal Transition
ER-α	estrogen receptor-alfa
Evs	Extracellular Vesicles
FBGC	Foreign Body Giant Cells
FOXO3	Forkhead box Other 3
GalNac	N-Acetilgalattosammine
GD	Disialoganglioside
GMB	Glioblastoma Multiforme
HCC	Hepatocellular Carcinoma Cells
HGNPs	NIR responsive hollow gold nanoparticle
HmgA2	High-mobility group AT-hook 2
HNSCC	Head and Neck Squamous Cell Carcinoma
KIP1	Kinesin-like protein 1
Lac-GLN	Lactosylated Gramicidin-Containing Lipid Nanoparticles
LMWP	Low Molecular Weight Protamines
LNA	Locked Nucleic Acid
LPD	Liposome-Protamine-DNA
LPH	Liposome-Polycation-Hyaluronic acid
LVs	Lentivirus
miRNA	microRNA
MM	Multiple Myeloma
NIR	Near-Infrared-Radiation
NOD	Non-obese diabetic
NPs	Nanoparticles
NSCLC	Non-Small Cell Lung Cancer
PAMAM	Poly Amidoamine
PDCD4	Programmed cell death protein 4
PDGFRa	Platelet-derived growth factor receptor-a
PDGFRb	Platelet-derived growth factor receptor-b
PEG	Polyethylene Glycol
PEI	Polyethylenimine
pHLIP	pH-induced transmembrane structure
PLGA	Poly Lactic-co-Glycolic Acid
PNAs	Peptide Nucleic Acids
PSMA	Prostate-Specific Membrane Antigen
PTEN	Phosphatase and tensin homolog
RDG	Arg-Gly-Asp
RECK	Reversion-inducing-cysteine-rich protein with kazal motifs
RES	Reticuloendothelial System
RVG	Rabies Virus Glycoprotein
SAE	Severe Adverse Events
scFv	Single-chain antibody Fragment
SCID	Severe combined immunodeficiency
SELEX	Systematic Evolution of Ligands by EXponential enrichment
SNALPS	Stable-Nucleic-Acid-Lipid-Particles
sncRNA	small non-coding RNA
SPRY1	Sprouty1
ssDNA	single-stranded DNA
ssRNA	single-stranded RNA
TIMP3	Metalloproteinase inhibitor 3
TNBC	Triple-Negative Breast Cancer
TRAIL	TNF-related apoptosis-inducing ligand
UTRs	Untranslated Regions
VEGFR	Vascular Endothelial Growth Factor Receptor
ZEB1	Zinc finger E-box-binding homeobox 1
ZEB1	Zinc finger E-box-binding homeobox 2
